# Moderating factors for the effectiveness of group art therapy for schizophrenia: secondary analysis of data from the MATISSE randomised controlled trial

**DOI:** 10.1007/s00127-014-0876-2

**Published:** 2014-04-11

**Authors:** Baptiste Leurent, Helen Killaspy, David P. Osborn, Mike J. Crawford, Angela Hoadley, Diane Waller, Michael King

**Affiliations:** 1 Research Department of Primary Care and Population Health, University College London, London, UK; 2 PRIMENT Clinical Trials Unit, University College London, London, UK; 3 Mental Health Sciences Unit, University College London, London, UK; 4 Camden and Islington NHS Foundation Trust, London, UK; 5 Centre for Mental Health, Imperial College London, London, UK; 6 School of Applied Social Science, University of Brighton, Brighton, UK; 7 London School of Hygiene and Tropical Medicine, London, UK

**Keywords:** Art therapy, Schizophrenia, Randomised controlled trial, Effect modifier, Subgroup analysis

## Abstract

**Purpose:**

Although some studies suggest that art therapy may be useful in the treatment of negative symptoms of schizophrenia, a recent large trial of group art therapy found no clinical advantage over standard care, but the study population was heterogeneous and uptake of the intervention was poor. This study aimed to investigate whether art therapy was more effective for specific subgroups of patients.

**Methods:**

Secondary analysis of data from a randomised controlled trial of group art therapy as an adjunctive treatment for schizophrenia (*n* = 140) versus standard care alone (*n* = 137). Positive and Negative Syndrome Scale scores at 12 months were compared between trial arms. Interaction between intervention effect and different subgroups, including those with more severe negative symptoms of schizophrenia, and those who expressed a preference for art therapy prior to randomisation, was tested using a linear mixed model.

**Results:**

The clinical effectiveness of group art therapy did not significantly differ between participants with more or less severe negative symptoms [interaction for difference in PANSS = 1.7, 95 % CI (−8.6 to 12.1), *P* = 0.741], or between those who did and did not express a preference for art therapy [interaction = 3.9, 95 % CI (−6.7 to 14.5), *P* = 0.473]. None of the other exploratory subgroups suggested differences in intervention effect.

**Conclusions:**

There was no evidence of greater improvement in clinical symptoms of schizophrenia for those with more severe negative symptoms or those with a preference for art therapy. Identification of patients with schizophrenia who may benefit most from group art therapy remains elusive.

## Background

Schizophrenia is a severe mental illness affecting up to one in a hundred people at some point in their lives. As well as positive symptoms, such as hallucinations and delusions, many people also experience negative symptoms, such as apathy and reduced organisational skills that can greatly impair their everyday functioning [1]. Art therapy, a form of psychotherapy which uses the medium of art to facilitate personal expression and understanding of emotions [2], has been shown through one exploratory trial to be associated with improvement of negative symptoms of schizophrenia (3) and, along with other arts therapies, is included in [3] the United Kingdom (UK) National Institute for Health and Clinical Excellence (NICE) guidelines for the treatment of schizophrenia [4]. However, a recent large pragmatic randomised controlled trial, the “MATISSE” study (Multicentre study of Art Therapy In Schizophrenia; Systematic Evaluation), found no evidence of a population-level effect of group art therapy over treatment as usual in terms of global functioning or symptoms of mental illness [5]. Nevertheless, results from a qualitative sub-study conducted alongside the MATISSE trial suggested that some patients reported benefits from the intervention, such as improvement in self esteem and social confidence [6]. Identifying subgroups of patients who are most likely to benefit from an intervention is obviously important when resource constraints limit its provision. However, large datasets are required to identify factors predictive of better outcome (such as patient characteristics). The MATISSE study included over 400 participants with a diagnosis of schizophrenia, an adequate sample size to explore potential predictors of difference in effectiveness. Based on previous research, two factors appear to be of particular interest: the severity of negative symptoms experienced [3, 4]; and having a preference for art therapy. One possible explanation for the lack of effectiveness reported in the MATISSE trial was the low uptake of art therapy groups [7]. The trial was pragmatic in nature and included a heterogeneous group of participants. Beyond a general willingness to be randomised to one of the trial arms and adhere to the allocated treatment, no specific account was taken of participants’ interest in art therapy before recruitment and randomisation. Participant preference for the interventions offered in a randomised trial may influence recruitment, attrition and adherence [8, 9]. It follows that those who are randomised to receive their treatment of choice may derive greater benefits than those with little interest in it, possibly through higher adherence to the intervention. It is also possible that those who were more comfortable talking about their feelings and more interested in creative arts may have derived more benefit from the art therapy groups. Similarly, those who are generally more adherent with their mental health treatment may have been more likely to engage constructively with the intervention. Finally, the participants recruited into the MATISSE trial had a median duration of illness of 15 years and it may be that the intervention has greater effectiveness at an earlier stage of the illness.

In this study, we sought to investigate the hypotheses that the clinical effectiveness of group art therapy delivered in the MATISSE trial was related to (a) the severity of negative symptoms of schizophrenia and (b) having a preference for the art therapy intervention. We also explored other related participant characteristics for their association with the effectiveness of the intervention: gender, adherence with current treatment and support; degree to which they felt comfortable talking about their feelings; interest in creative arts; and length of contact with mental health services.

## Methods

This paper reports results of a secondary analysis of data collected in the MATISSE randomised controlled trial (ISRCTN46150447). The trial protocol and main results are reported elsewhere [10, 11].

### MATISSE trial overview

The objective of the MATISSE trial was to evaluate the clinical and cost-effectiveness of interactive group art therapy for patients with a diagnosis of schizophrenia. After recruitment, participants were randomly allocated to one of three arms: treatment as usual; treatment as usual plus activity group; treatment as usual plus interactive group art therapy. The activity group provided an “attention control” arm to allow for the effect of attending a group activity and is not part of this secondary analysis. Participants were interviewed at recruitment, 12 and 24 months using standardised measures to assess symptoms of schizophrenia, global functioning, satisfaction with care, engagement with treatment and social functioning. The trial found that the addition of art therapy to usual care resulted in no clinical advantage over usual care alone or usual care plus activity groups and was not more cost-effective [5].

### Settings and participants

Participants were recruited in 15 community based secondary mental health and social care services in four centres across UK (West London, North London, Avon and Wiltshire, and Belfast). Inclusion criteria included being at least 18 years old and having a clinical diagnosis of schizophrenia confirmed by operationalised criteria using case note review [12]. Those whose mental health problems meant they lacked capacity to be able to give informed consent to participate were excluded, as were those unable to speak sufficient English to complete baseline assessment, and those currently receiving any form of arts therapy (art, drama, dance, music, body psychotherapy). All participants had to be willing to take part in art therapy or activity groups and all provided written informed consent to participate in the trial.

### Intervention

Those randomised to art therapy were offered weekly sessions of 90 min for an average period of 12 months. Group art therapy was conducted in keeping with the recommendations of the British Association of Art Therapists [13] and reflected usual group art therapy delivered in the UK National Health Service. All groups were facilitated by art therapists registered with the Health Professions Council with previous experience of working with people with psychosis, and co-facilitated by another member of staff. A range of art materials were available and participants were encouraged to use them to express themselves freely and spontaneously. Art therapists generally adopted a supportive approach, offering empathy and encouragement; they rarely provided symbolic interpretations of interpersonal process or images to participants. Within this framework, therapists used a range of interventions thought appropriate to each participant. All art therapists received monthly supervision from a senior art therapist in each centre. Senior members of the study team assessed the degree to which art therapy was delivered in accordance with the study protocol guidance by reviewing proforma completed by the art therapists after each session.

Treatment as usual comprised access to standard care from secondary mental health services, including care coordination, pharmacotherapy, and referral to other therapies as clinically indicated, with the exception of arts therapies.

### Measures

The Positive and Negative Syndrome Scale (PANSS) [14] is a standardised measure of symptom severity for patients with schizophrenia. It comprises 30 items completed through a structured interview with the researcher. Each item is scored from 1 (pathology absent) to 7 (extreme pathology), and items are summed to give a total score from 30 to 210. The scale can be divided into three subscales: positive, negative and general symptoms of schizophrenia.

The Morisky scale [15] is a four-item self-report scale that assesses participants’ non-adherence to medication. Items are rated from 0 to 4, with four indicating poorer adherence.

The Engagement and Acceptance Scale (EAS) [16] is a four-item scale completed by the participant’s care co-ordinator that assesses the degree to which the participant is engaged with mental health services. Each item is rated from 0 to 4 with a total score from 0 to 16, with higher scores reflecting greater engagement.

 At baseline, participants were asked whether they had a preference for any one arm of the study (treatment as usual, activity group, art therapy group, or no preference). They were advised that their answer would not affect the outcome of randomisation. Two additional questions were also asked at the baseline interview, each rated from 1 to 5. The first assessed participants’ views of creative activities (1—I like being creative and make opportunities to do creative things in everyday life; 5—I avoid doing creative things). The second assessed the degree to which they felt at ease talking about thoughts and feelings (1—I am very comfortable describing what I think and feel; 5—I am unable to describe what I think and feel).

### Subgroups

Subgroups were defined a priori, according to clinical relevance or to median values of assessment measures. Two subgroups were defined to test our primary hypotheses and the others were used to explore other possible interactions.

In keeping with previous literature [17, 18], a score of 20 or more on the negative symptoms subscale of the PANSS was used to define participants with a high severity of negative symptoms. For our preference subgroup analysis, participants who expressed a preference for art therapy at recruitment were compared to those who reported a preference for other trial arms or no preference.

Other subgroups were defined as follows; those with less than and more than 10 years contact with mental health services; those with good adherence to medication (Morisky scale score 0) and poor adherence (Morisky scale score 1–4); those with greater and poorer engagement with services (above and below the Engagement and Acceptance Scale median score of 12); those who liked creative activities (score 1 or 2 on our scale) and those who did not (score 3–5); those who were more at ease talking about their feelings (score 1 on our scale) and those who were not (score 2–5).

### Statistical analysis

A detailed statistical analysis plan was developed before data analysis commenced and is available online [19]. Participants were compared according to their allocation arm, independently of their adherence with the intervention (intention-to-treat). When comparing proportions, Pearson’s Chi-squared and Fisher’s exact tests were used as appropriate. The main analysis compared PANSS score at 12 months using a mixed-effect linear model, adjusting for baseline PANSS and recruitment site (random effect). The analysis was first carried out stratifying by subgroup of interest to estimate the intervention effect within each subgroup, and reported graphically in a forest-type plot. The moderative effects of the subgroups on the outcome were then tested by including an interaction term (subgroup by randomisation arm). A significant interaction parameter indicates that the effect of the intervention is not the same in each subgroup and that the subgroup is a “modification factor” for the effectiveness of the intervention. For continuous variables, the presence of a linear interaction (variable by randomisation arm) was also tested. Time since diagnosis was also log-transformed to test for a possible non-linear interaction. Regarding missing data, subgroups were only defined based on observed data. For the outcome (PANSS) at 12 months and at baseline, if a majority of the syndromes had been assessed and few (<10 %) were missing, they were imputed by regression imputation. If more than 10 % of items were missing, the total PANSS was considered as missing. Attrition and reasons for missingness were compared between arms.

All statistical tests are two-sided, and significance considered at the 5 % level. All differences between trial arms are reported for art therapy compared to treatment as usual. The statistical software Stata (version 12, for Windows) was used for all analyses.

## Results

### Participants

**Fig. 1:**
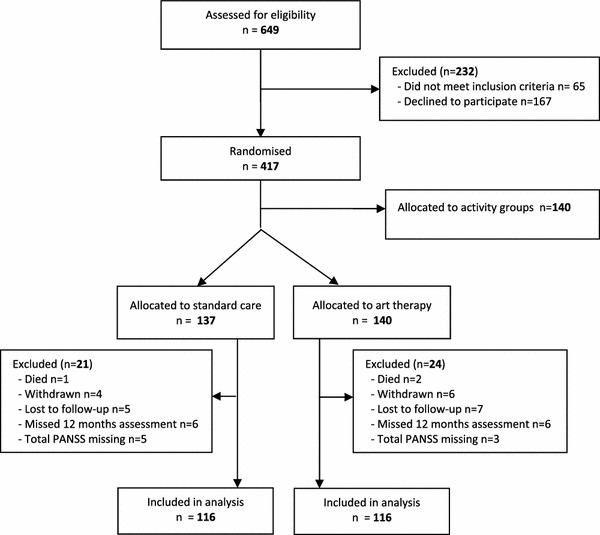
Study flow chart

A total of 649 patients from 15 participating sites were assessed for eligibility and 417 were enrolled into the trial, including 277 randomised to either treatment as usual (*n* = 137) or art therapy (*n* = 140) (Fig. 1). A total of 45 randomised participants (16 %) could not be included in the analysis for various reasons (see Fig. 1). The attrition rates at 12 months and the reason for attrition did not significantly differ by arm [*χ* ^*2*^(*1*) = 0.17, *P* = 0.68 and Fisher’s exact *P* = 0.65, respectively]. Attrition rates varied by study sites (Fisher’s exact *P* < 0.001), but no other baseline characteristics were significantly related to attrition. The amount of missing data was low: nine participants (3.2 %) had some of their PANSS items at baseline or follow-up imputed for the analysis.

Participants had a median age of 41 years old, and 68 % were male. The median duration of illness was 15 years, 32 % had been admitted to an inpatient psychiatric unit in the previous 12 months, and 95 % were prescribed antipsychotic medication. Some participants had previous experience of art therapy (29 %), and 11 % had experienced another form of creative therapy.

### Attendance

Overall, attendance to the art therapy sessions was poor, with 39 % of those allocated not attending any sessions, and a median number of 11 sessions attended for the remainder over the 12 month intervention period (range 1–51).

The proportion of participants who attended at least two sessions of art therapy is reported for each subgroup in Table 1. There was a suggestion of higher attendance in patients who had expressed a preference for art therapy when they were recruited [69 vs. 51 %, *χ* ^*2*^(*1*) = 3.2, *P* = 0.073], and for those who reported that they were comfortable speaking about their thoughts and feelings [64 vs. 47 %, *χ* ^*2*^(*1*) = 2.7, *P* = 0.098].

**Table 1:** Attendance and outcome by subgroup of participants

Subgroup	Frequency *n* (%)	Attendance ≥2 sessions *n* (%)^a^	*P* value attendance	PANSS score at 12 months Mean (SD)		Adjusted^b^ difference (95 % CI)	*P* value subgroup interaction	*P* value linear interaction
				TAU	ART			
Overall	232 (100)	67/116 (58)		70.9 (24.5)	72.7 (27.1)	−0.3 (−5.4 to 4.8)		
Primary subgroups								
Negative symptoms								
No (<20)	136 (59)	37/65 (57)	0.740	61.9 (19.7)	62.0 (17.3)	−0.6 (−6.5 to 5.2)	0.741	0.437
Yes (≥20)	95 (41)	30/50 (60)		85.2 (24.8)	87.0 (31.0)	0.5 (−8.4 to 9.4)		
Baseline arm preference								
Preference for ART	93 (43)	27/39 (69)	0.073	73.2 (25.8)	76.6 (30.6)	2.5 (−6.0 to 10.9)	0.473	–
Other	125 (57)	35/68 (51)		67.9 (23.1)	70.2 (25.1)	−1.2 (−7.9 to 5.5)		
Exploratory subgroups								
Gender								
Male	158 (68)	40/72 (56)	0.539	70.6 (22.5)	71.0 (25.2)	−0.9 (−6.9 to 5.1)	0.686	–
Female	74 (32)	27/44 (61)		72.0 (30.1)	75.4 (30.1)	1.0 (−8.7 to 10.7)		
Time since diagnosis								
<10 years	46 (21)	16/23 (70)	0.234	75.8 (28.1)	74.1 (24.8)	−2.5 (−14.6 to 9.5)	0.756	0.700
≥10 years	172 (79)	48/86 (56)		70.6 (23.9)	72.2 (28.6)	−0.8 (−6.6 to 5.1)		
Adherence to medication								
Good (Morisky = 0)	114 (50)	34/60 (57)	0.780	68.3 (23.8)	71.9 (26.9)	0.3 (−7.0 to 7.6)	0.768	0.614
Poor (Morisky ≥ 1)	113 (50)	32/54 (59)		73.5 (25.2)	72.4 (27.2)	−1.4 (−8.8 to 6.0)		
Engagement and acceptance								
Poor (EAS < 12)	82 (51)	22/41 (54)	0.927	80.7 (23.7)	83.5 (33.7)	−3.0 (−12.5 to 6.4)	0.786	0.600
Good (EAS ≥ 12)	78 (49)	20/38 (53)		69.6 (26.3)	68.6 (23.9)	−0.9 (−10 to 8.2)		
Creative activities								
Enjoy more (<3)	153 (67)	45/71 (63)	0.250	68.1 (25.4)	69.4 (26.6)	−0.6 (−6.8 to 5.7)	0.913	0.580
Enjoy less (≥3)	74 (33)	22/42 (52)		77.5 (21.1)	77.6 (28.3)	0.2 (−9.2 to 9.6)		
Talking about thoughts								
Comfortable (<2)	82 (36)	16/34 (47)	0.098	71.7 (26.9)	68.2 (26.1)	0.9 (−7.3 to 9.1)	0.894	0.672
Uncomfortable (≥2)	146 (64)	51/80 (64)		70.1 (22.9)	74.4 (27.7)	0.1 (−6.7 to 6.8)		

*ART* art therapy arm, *TAU* treatment as usual arm, *SD* standard deviation, *EAS* Engagement and Acceptance Scale

^a^Intervention arm only. %, proportion of patients in this subgroup in the intervention arm who attended at least two sessions of art therapy

^b^Difference between trial arms, adjusted for baseline PANSS score and clustering by site

### Relationship between subgroups and effectiveness of art therapy

**Fig. 2:**
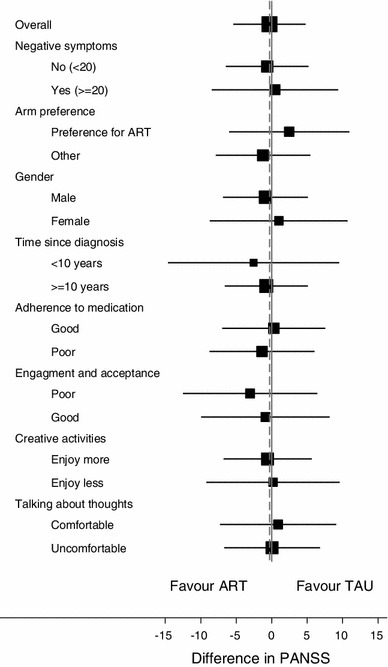
Forest plot of mean difference between arms in PANSS at 12 months, by subgroup of participants. Estimated mean difference (and 95 % CI) in PANSS score at 12 months, adjusted for baseline PANSS and site. The size of the square is proportional to the number of observations. The *solid vertical line* represents zero difference, and the *dashed vertical line* the overall mean difference. *ART* art therapy arm, *TAU* treatment as usual arm

The estimated effect of art therapy, according to the subgroups of interest, is reported in Table 1 and plotted in Fig. 2. In the subgroup of participants with more severe negative symptoms, the adjusted mean difference between arms in PANSS at 12 months was of 0.5 points [95 % confidence intervals (CI) (−8.4 to 9.4)]. The effect of the intervention did not differ between patients with or without severe negative symptoms [interaction = 1.7, 95 % CI (−8.6 to 12.1), *χ* ^*2*^(*1*) = 0.11, *P* = 0.741]. Similarly, when testing for the PANSS negative subscale score as a linear modification factor, the interaction term was not significant [interaction = 0.3, 95 % CI (−0.4 to 1.0), *χ* ^*2*^(*1*) = 0.60, *P* = 0.437], which suggests that the effectiveness of the intervention was not associated with the severity of negative symptoms as assessed at baseline.

In the subgroup of participants who expressed a preference for art therapy at recruitment, those in the intervention arm had on average a 2.5 higher PANSS score at 12 months [95 % CI (−6.0 to 10.9)], and there was no significant difference in effect between those with or without a preference for art therapy [interaction = 3.9, 95 % CI (−6.7 to 14.5), *χ* ^*2*^(*1*) = 0.51, *P* = 0.473].

None of the other exploratory subgroups showed significant differences in intervention effect [*χ* ^*2*^(*1*) range: 0.01–0.31, *P* range: 0.580–0.914] (Table 1).

## Discussion

We found no difference in the clinical effectiveness of group art therapy in participants with more severe negative symptoms, or between those with and without a preference for art therapy. We found no significant moderating effect on art therapy effectiveness of the other participant characteristics we explored.

In its 2009 guidelines on the management of schizophrenia [4], NICE recommended offering arts therapies, particularly to people with negative symptoms. This recommendation was based on the 2009 guidelines (reference: NICE (2009)), which reviewed six randomised trials of arts therapies. The trials were of varying quality, but generally suggested that creative therapies were associated with reduction of negative symptoms [4]. Most of these trials investigated music therapy, but one exploratory trial of art therapy also showed an effect on negative symptoms [3]. Negative symptoms of schizophrenia may be associated with difficulty engaging in psychological therapies, and art therapy may therefore be a suitable non-verbal alternative for such patients. In theory, it might be expected that interventions based on non-verbal expression that include a social interaction element, such as interactive group art therapy, could impact positively on the negative symptoms of schizophrenia such as poor social rapport and emotional withdrawal. However, the main analysis of the MATISSE randomised trial did not find any effect of art therapy on negative symptoms at 12 and 24 months. The present analysis has further corroborated this finding: art therapy was not found to be more effective for those with more severe negative symptoms. There are a number of reasons why the findings from the MATISSE study may have differed from those reported by Richardson et al. Firstly Richardson’s study was an exploratory trial with high attrition and multiple outcomes. The difference observed between groups in ratings of negative symptoms was around statistical significance level and may have been observed by chance alone. Secondly, the form of art therapy delivered may have differed from that delivered in the MATISSE study, although they were both based on similar guidelines [13, 20, 21]. Finally, outcomes in the MATISSE study were assessed longer after recruitment (12 months) than in the Richardson et al. study (6 months) and thus, any initial clinical gains noted by Richardson et al. may have dissipated over time.

Our second hypothesis explored the possibility that art therapy may be more effective in patients who expressed a preference for the intervention and might therefore be more likely to engage with it. MATISSE was a pragmatic trial, and included participants who may not have been enthusiastic or knowledgeable about art therapy. Restricting the analysis to those who expressed a preference for art therapy potentially offers a better estimate of the efficacy of the intervention in those willing to engage with it. We found that participants who expressed a preference for art therapy appeared to have a slightly higher attendance but this did not result in greater benefit. This finding is consistent with a systematic review of “preference trials” by King et al. [22] which showed little difference in effectiveness between those allocated to their arm of preference, and those randomly allocated. It may also be explained by the absence of any overall effect of art therapy in the main MATISSE study even after controlling for adherence to the intervention [5].

Eligibility criteria in the MATISSE study were broad. In view of the small effect observed when group art therapy was offered to all patients with a diagnosis of schizophrenia, and the resources involved in delivering such an intervention, it is important to identify patients who appear the most likely to benefit from it [6]. Subgroup analysis can also shed light on the therapeutic mechanisms of an intervention and generate hypothesis on why it may or may not have work. We therefore performed exploratory analyses to investigate whether other participant characteristics were associated with the effectiveness of the intervention. However, we did not identify any subgroups of participants for whom art therapy appeared more effective. These findings concur with the main findings of the MATISSE study.

The MATISSE study is the largest randomised evaluation of art therapy for schizophrenia to date and provided sufficient data to investigate potential heterogeneity of the effect of art therapy between subgroups. The main pitfall of subgroup analysis is the increased risk of chance findings when multiple unjustified comparisons are performed [23, 24]. This risk was reduced by restricting the number of subgroups, and making the distinction between our two primary hypotheses and further exploratory subgroups. Another risk of subgroup analysis is if researchers identify significant effect modifiers “by chance” during exploration of the data, and become more likely to publish this finding. This is why it is essential for subgroup analyses to be specified a priori and to report all analyses performed [23, 24]. Although this secondary analysis was not planned as early as the trial design stage (and with knowledge of the main trial result), the analysis plan and scale cut-offs were agreed before any exploration of possible effect modifiers. The analysis plan [19] was then made publicly available before commencing the analysis, to provide transparency and prevent selective reporting. However, as the trial was not designed to test for interactions, these secondary analyses may have been under powered, since such analyses require larger sample sizes, and non-statistically significant effects do not demonstrate the absence of interactions. Nevertheless, the sample size is sufficient to rule out any important differences between subgroups. The general limitations of the MATISSE trial, as reported in Crawford et al. [5], also apply to this analysis: the use of ‘closed’ art therapy groups set up specifically for the trial could explain the low attendance, even in those who expressed a preference for art therapy; the small size of the groups, which may have limited social interactions between participants; the intervention may have offered benefits in the shorter term, or on other outcomes valued by the patients not assessed in the study [6]. The exploratory analyses performed have further limitations, for example non-standardised single item Likert-type scale was used to assess the degree to which participants felt comfortable talking about their feelings and their interest in creative arts. Also, although no interaction with time of illness was seen overall, due to small numbers in this subgroup, it was not possible to asses if those recently diagnosed (e.g. within 6 months) could have shown greater benefits.

## Conclusion

This study did not demonstrate greater clinical benefit from group art therapy for people with more severe negative symptoms of schizophrenia, or for those who expressed a preference for it. In view of the potential benefits but conflicting evidence, further studies of creative therapies for the treatment of schizophrenia are indicated.
